# Antinuclear Antibody (ANA) and Anti-Mi-2-Alpha Positive Dermatomyositis Hinting a Cancer Diagnosis

**DOI:** 10.7759/cureus.21844

**Published:** 2022-02-02

**Authors:** Mariana Pacheco, Helena Ferreira, Clara Silva, João Silva, Emanuel Matias, Teresa Antunes, Jorge S Almeida

**Affiliations:** 1 Internal Medicine, Centro Hospitalar Universitário de São João, Porto, PRT; 2 Medicine, Faculty of Medicine of the University of Porto (FMUP), Porto, PRT

**Keywords:** antinuclear antibody, adult onset, cancer, anti-mi2 alpha, dermatomyositis

## Abstract

Dermatomyositis (DM) is a relatively uncommon inflammatory myopathy that has been linked to cancer. We report the case of an 81-year-old woman with cecum adenocarcinoma presenting with antinuclear antibody (ANA) and anti-Mi-2-alpha antibody-positive DM. The patient complained of anorexia, symmetric proximal muscle weakness and skin rash and presented with elevated muscle enzymes. A skin and muscle biopsy supported the diagnosis of DM as did the limbs magnetic resonance imaging (MRI) and electromyography. A diagnosis of localized adenocarcinoma of the cecum was made through colonoscopy and the patient was successfully surgically managed, with decreasing muscle enzymes at discharge and gradual recovery of muscle strength. The presence of both ANA and anti-Mi-2 autoantibodies has classically been described as comprising a better prognosis with a lower risk of underlying malignancy.

This case highlights the importance of pursuing a cancer diagnosis in elderly patients presenting with DM even in presence of less predisposing immunological profiles.

## Introduction

Dermatomyositis (DM) is a relatively uncommon inflammatory myopathy that affects mostly females and can occur at any age [1]. The main clinical feature is symmetric proximal muscle weakness. It can be accompanied by muscle tenderness, but stiffness is usually not a prominent feature [1]. Skin involvement comprises Gottron’s papules (symmetrical erythematous papules over extensor bony prominences); Gottron’s sign; heliotrope/facial erythema that involves nasolabial folds; poikiloderma (hyper- and hypopigmentation) affecting sun-exposed areas (shawl and V-signs), lateral surfaces of the thighs (Holster’s sign); scaling of the scalp; and nail fold abnormalities [2]. A subgroup of patients can develop interstitial lung disease, oesophageal involvement (dysphagia), or cardiac involvement (myocarditis and/or conduction abnormalities) [1].

Diagnosis is based on clinical features, laboratory findings - myositis-specific and myositis-associated autoantibodies positivity, elevated serum creatine kinase (CK), lactate dehydrogenase (LDH) and/or transaminases (ALT/AST), and histologic findings on skin and muscle biopsy. Electromyography can be helpful in differentiating myopathies from neuropathies. Magnetic resonance imaging (MRI) can be helpful in cases of atypical clinical presentations and in selecting a biopsy site [1,3].

When a diagnosis is established, it is important to exclude lung involvement and to screen for malignancy [1,4,5]. DM is associated with an increased risk of cancer and in European countries, the most frequently reported cancers were lung, breast, ovarian, pancreatic and colorectal cancers [6]. Risk factors for malignancy include older age at disease onset, severe cutaneous disease, resistance to treatment, prior history of malignancy with risk of relapse and certain immunological profiles. The presence of myositis-specific autoantibodies such as anti-NXP-2 antibodies or anti-TIF-1gamma is associated with an increased risk of cancer [5,7,8].

Treatment involves high-dose glucocorticoids (1mg/kg per day of prednisone) for 4-6 weeks. For severely ill patients, IV methylprednisolone pulses can be considered. Slow tapering of corticosteroid to the lowest effective dose should be carried out for 9-12 months. Muscle enzyme normalization and muscle strength recovery are indicators of response to therapy [9]. Alternatively, treatment can be initiated with glucocorticoids in combination with glucocorticoid-sparing agents, namely azathioprine or methotrexate [10]. Notably, malignancy-associated DM may improve after treatment of cancer [11].

This report refers to an elderly patient with colon cancer-associated anti-Mi-2-alpha positive DM, with concurrent ANA positivity. It aims to review and discuss existing literature on the subject and to highlight the relevance of pursuing a diagnosis of underlying malignancy in all DM cases.

## Case presentation

An 81-year-old woman was referred to our hospital by her assistant physician due to anorexia, symmetric proximal muscle weakness and erythematous-desquamative skin rash involving the face (malar region, not sparing the nasolabial fold, and eyelids - heliotrope eruption) and extensor surfaces of arms, hands and legs (Gottron’s sign) for the previous month coupled with creatine kinase, myoglobin and transaminases elevation, in blood tests.

The patient's past medical history comprised arterial hypertension, dyslipidaemia and obesity. The patient denied knowledge of previous personal cancer history or infectious conditions and the patient’s family history was unremarkable. The patient was on atorvastatin 10mg per day, valsartan 160mg/hydrochlorothiazide 12.5mg per day, lansoprazole 30mg per day, alprazolam 0.5mg per day. No pharmacological allergies were known and no drugs, herbal remedies or teas had been introduced, recently.

The patient denied having had a fever, weight loss, arthralgia, articular swelling or pain. She did not report any other gastrointestinal (GI), genitourinary, respiratory or cardiovascular symptoms and denied xerostomia and xerophthalmia, as well as the mouth or genital ulceration. No past history of miscarriage or thrombosis was apparent. The environmental context was unworthy of note.

**Figure 1:**
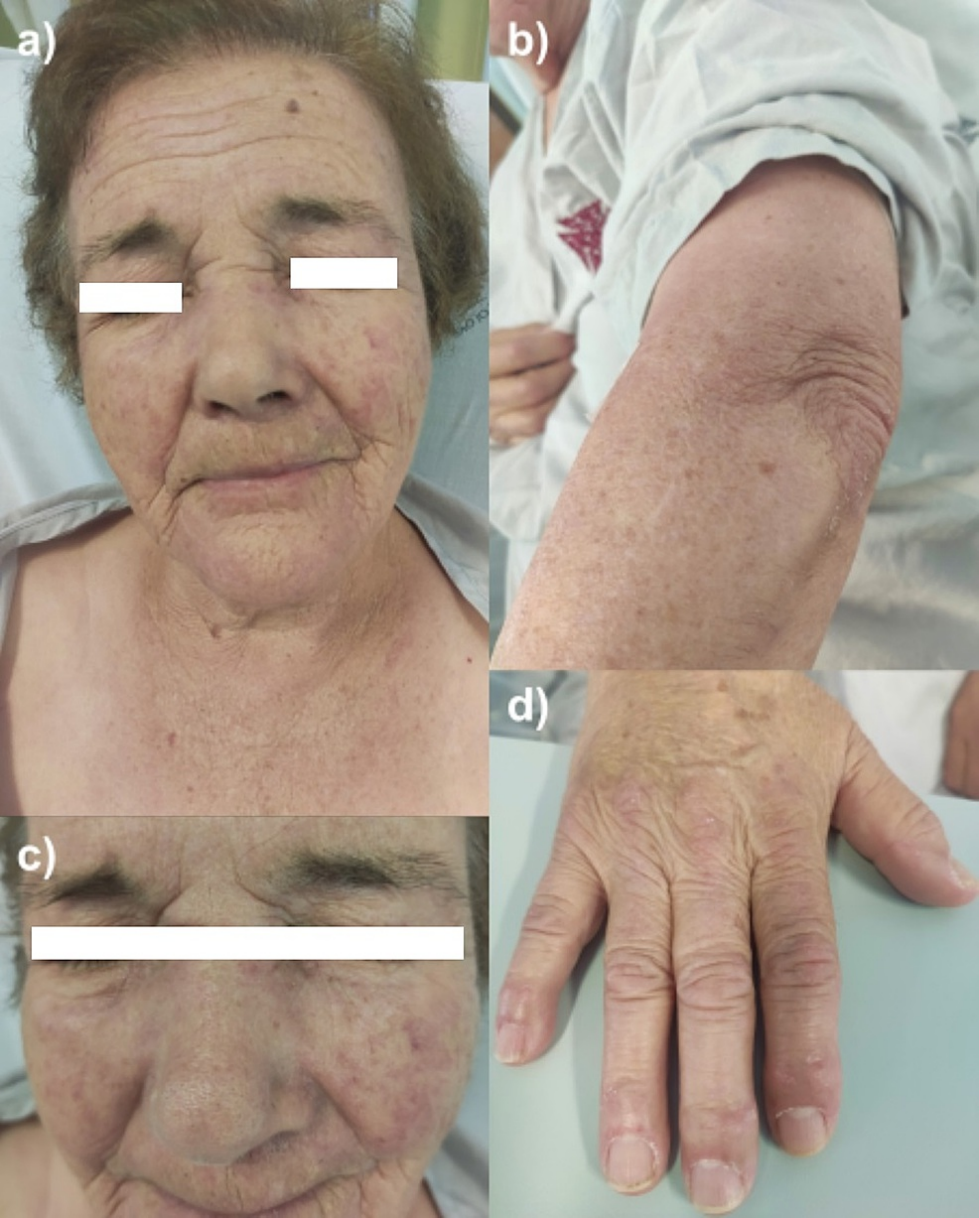
Dermatomyositis skin rash (a) V-sign and heliotrope/facial erythema that involves nasolabial folds. (b) Gottron sign: extensor surface of the elbow presenting erythematous and scaling plaques. (c) Facial erythema that involves nasolabial folds. (d) Gottron sign: erythematous patches on the extensor surfaces of the joints of the hands with periungual erythema.

On physical examination, blood pressure was 128/94 mmHg, heart rate 80 bpm with a rhythmic pulse, respiratory rate 16/min and temperature 36ºC. The air oxygen saturation was 98%. No anomalies were found in cardiopulmonary auscultation and the abdominal examination was unremarkable. Slight peripheral oedema was apparent and capillary perfusion time was <2sec. Neurological examination at admission was normal. The patient was not able to elevate her arms above a 60-degree angle and could not stand up from a sitting position, on her own. After assuming an orthostatic position, the patient could walk without support for short distances. Muscle palpation and dimension were normal, with no external signs of trauma or painful areas. A skin rash was apparent as described previously and displayed in Figures 1a-1d.

Investigation

The most relevant performed laboratory studies and normal reference values are detailed in Table 1 and discussed in the following paragraphs.

**Table 1:** Summary of laboratory findings

Parameter	Value		Normal range	
Haemoglobin	13.8	g/dL	12.0-16.0	g/dL
MCV (mean corpuscular volume)	85.2	fL	87-103	fL
MCHC (mean corpuscular haemoglobin concentration	32.8	g/dL	28-36	g/dL
Leucocytes	6.14	x10^9 /L	4.0-11.0	x10^9 /L
Platelets	225	x10^9 /L	150-400	x10^9 /L
ESR (erythrocyte sedimentation rate)	14	mm/1^st^ hour	0-30	mm/1^st^ hour
CRP (C-reactive protein)	3.7	mg/L	<3.0	mg/L
ALT (alanine aminotranferase)	145	U/L	10-31	U/L
AST (aspartate aminotransferase)	138	U/L	10-31	U/L
GGT (gamma-glutamyltransferase)	22	U/L	7-32	U/L
Alkaline phosphatase	53	U/L	30-120	U/L
Total bilirubin	0.69	mg/dL	<1.20	mg/dL
Urea	41	mg/dL	10-50	mg/dL
Creatinine	0.40	mg/dL	0.51-0.95	mg/dL
Creatine kinase	2,779	U/L	10-149	U/L
Myoglobin	3,819.6	mg/dL	<146.9	mg/dL

The complete haemogram and coagulation panel were normal. Serum urea, serum creatinine and electrolyte panel showed no abnormalities. Lactate dehydrogenase was elevated (595U/L) with only a slight elevation of C-reactive protein (3.5mg/L). Laboratory investigation confirmed increased and rising creatine kinase (1776U/L up to a maximum of 3037U/L) as well as steady mild elevation of AST/ALT. Albumin, total and direct bilirubin as well as γ-glutamyl transferase and alkaline phosphatase were normal.

Using the clinical and analytical data available up to this point, according to EULAR/ACR criteria for idiopathic inflammatory myopathies our patient was already classified as having a 100% probability for a diagnosis of DM, reinforcing the prominence of the clinical aspects in recognition of these entities [12].

Further testing found no abnormalities in HbA1c, thyroid function or vitamin B12 and folic acid levels. Serum iron as transferrin saturation was low with a ferritin of 98ng/mL. The urinalysis showed microalbuminuria, slight leukocyturia (80/µL) and no pathological casts.

Anti-Mi-2-alpha antibodies were positive as were ANA (titre 1/1,000). The remaining myositis-specific panel, myositis-associated panel and anti-ENA antibodies were negative. ANCA (PR3 and MPO), anti-DsDNA, anti-cardiolipin, rheumatoid factor, anti-CCP and circulating immune complexes titres were negative. Complement fractions C3c and C4 were within normal levels. Electrophoresis of the serum proteins demonstrated a normal profile and immunoglobin levels and light chain proteins in the serum were within normal values. Beta-2 microglobulin was mildly elevated (3,050µg/L), due to the inflammatory context. TPPA and HIV, hepatitis B virus (HBV) and hepatitis C virus (HCV) serology were negative.

**Figure 2:**
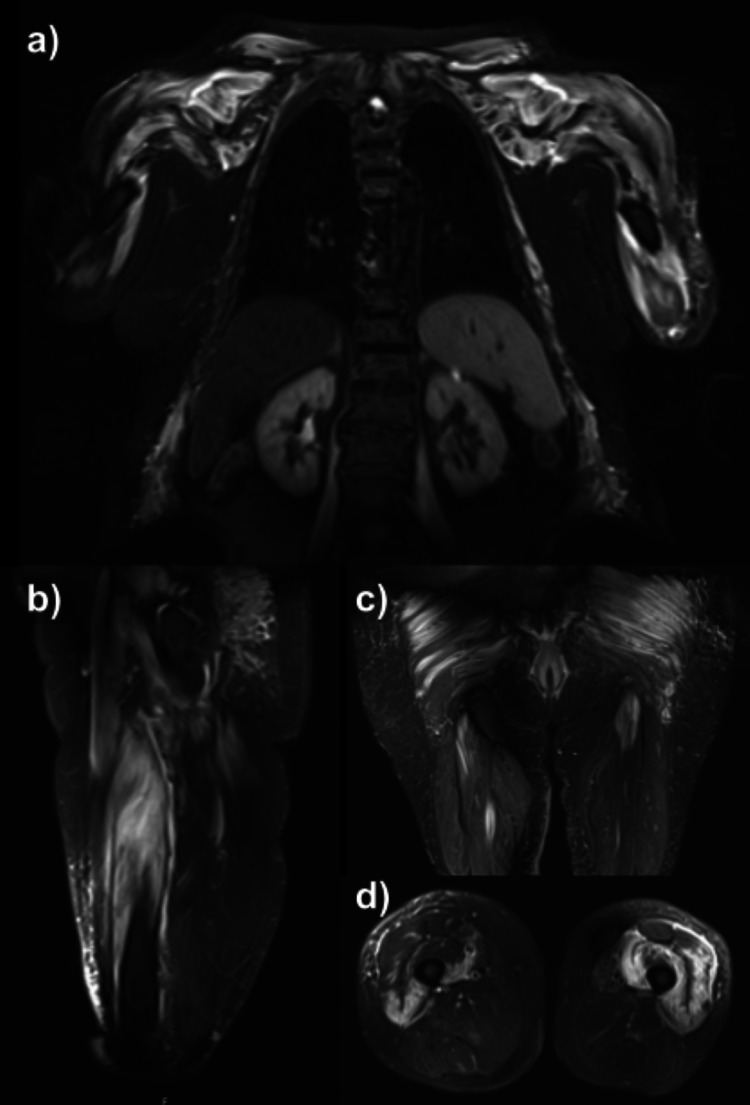
MRI - T2-weighted images presenting hyperintense signal throughout the affected muscles and peri-muscular oedema (a) Periscapular muscles in coronal view. (b) Thigh muscles in sagittal view. (c) Hip muscles in coronal view - gluteal aspect. (d) Thigh muscles in axial view.

MRI of the legs and arms showed marked inflammatory changes involving paraspinal muscles and both upper and lower limb proximal muscles with subcutaneous tissue oedema, suggesting a diffuse inflammatory process (Figures 2a-2d).

Skin biopsy of the arm displayed vacuolar changes and apoptosis of basal keratinocytes with mononuclear perivascular infiltrates in the superficial dermis as well as interstitial mucin deposition, pointing to either DM or acute cutaneous lupus. After performing the skin biopsy, the patient was started on topical corticosteroids with reduction of the erythema and scaling of the lesions. The images in Figure 1 were obtained after corticosteroid initiation and are, therefore, not as exuberant as presented at admission.

Electromyography was compatible with DM as it suggested a myopathic pattern. Pulmonary function tests were performed to screen for lung involvement and were within normal range, with normal diffusing capacity for carbon monoxide (DLCO). A CT scan of the neck, thorax, abdomen, and pelvis was performed to screen for malignancy and disclosed no relevant findings. Further work-up comprised a mammogram and breast ultrasound as well as GI tract endoscopies. The mammogram and breast ultrasound scored BIRADS-2. Upper GI endoscopy showed congestive gastropathy with gastric biopsies describing areas of chronic gastritis. Colonoscopy showed a 25mm vegetating lesion in the caecum suggestive of neoplasia. Biopsy confirmed adenocarcinoma of the colon.

An eco-guided muscle biopsy showed muscle fibre necrosis and perifascicular atrophy, reinforcing the diagnosis of antiMi-alpha2 positive DM as a secondary diagnosis that led to the unveiling of localized adenocarcinoma of the cecum as the primary condition.

Treatment

The patient was discussed in a multidisciplinary oncology meeting and referred for a right hemicolectomy, addressing the tumour. The surgery occurred without complications. A discreet improvement of muscle strength (patient able to reach the head with both hands and stand up without help) and descending CK serum levels (1,000U/L at discharge) was noted post-operatively. Further need for adjuvant immunosuppressant therapy following surgery will be ascertained during follow-up, according to the clinical and analytic responses. The patient was advised to promptly seek medical attention if abnormal symptoms presented or known symptoms worsened.

## Discussion

An association between inflammatory myopathies (particularly DM) and cancer has been well documented with major series describing 6.7% to 32% of cases in the presence of an underlying oncologic condition [13]. The most frequent malignancies in this particular context appear to be similar to the general population. A diagnosis of inflammatory myopathy is reported to be simultaneous or to precede the diagnosis of cancer in up to a year, in most cases [14].

Myositis-specific autoantibody profile can relate to clinical features and distinct inflammatory syndromes, supporting an influence on prognosis. The prevalence of anti-Mi-2 autoantibodies ranges from 2% to 38% in adults with DM [13,14]. Contrarily to anti-TIF-1gamma or anti-NXP-2 antibodies, the presence of anti-Mi-2 antibodies has classically been thought to predict a lower incidence of malignancy, better treatment response and, therefore, better outcome [7,8]. There is, however, one recent French series that displays conflicting conclusions describing the high risk of cancer in this subset of patients, when compared to the general population. In this study, no information about the anti-Mi-2 antibody isoforms (alpha or beta) tested for was available, according to the authors [15]. A Dutch study also suggested an increased risk of cancer in patients with antibodies to the N-terminal fragment of the anti-Mi-2-beta antigen. The anti-Mi-2-alpha isoform was not focused on, in this study [16]. This matter may require further investigation to standardize testing in order to accurately establish the prognostic value of the anti-Mi-2 antibody and its two isoforms, in this population. For the time being, most of the available evidence points to a lesser risk of cancer and better prognosis in patients with anti-Mi-2 associated DM. Furthermore, considering ANA in relation to DM, some studies report a negative correlation between ANA positivity and risk of cancer [17]. 

In the presence of a DM case with both ANA and anti-Mi-2-alpha antibodies positivity, a diagnosis of cancer in our patient would be a less expected occurrence, regarding the above-stated.

## Conclusions

This is, to our knowledge, the first reported case of colon cancer-related anti-Mi-2-alpha positive DM, with concurrent ANA positivity. It intends to highlight the importance of pursuing a cancer diagnosis, particularly in elderly patients with DM, even in the presence of less characteristic immunological profiles. Clinical follow-up and active cancer screening are recommended for three to five years following onset of DM if a concurrent malignancy is not identified at baseline. An accurate and timely diagnosis is of major relevance in assuring the best possible treatment and can impact survival.
